# Low-dose mivacurium facilitates laryngeal mask airway insertion in patients undergoing hysteroscopic surgery: a prospective, single-center, double-blind randomized controlled trial

**DOI:** 10.3389/fphar.2025.1700175

**Published:** 2025-10-17

**Authors:** Yingchao Guan, Lizhen Wu, Haochen Wang, Conghui Wang, Yusong Lin, Minghong Ju, Xiaojing Cong, Wen He, Xiaodong Wang

**Affiliations:** Department of Anesthesiology, Weihai Municipal Hospital, Cheeloo College of Medicine, Shandong University, Weihai, Shandong, China

**Keywords:** laryngeal mask airway, mivacurium, postoperative sore throat, postoperative nausea and vomiting, randomized controlled trial

## Abstract

**Background:**

To explore whether the application of mivacurium can facilitate laryngeal mask airway (LMA) insertion and benefit patients.

**Methods:**

A total of 167 patients undergoing hysteroscopy were randomly divided into mivacurium (group M) and control (group C) groups. The anesthesia induction scheme was mivacurium + sufentanil + propofol in group M, whereas mivacurium was replaced with saline in group C. The main outcome was the LMA insertion condition Secondary outcomes included attempts and elapsed time of LMA insertion, intraoperative anesthetic consumption, perioperative hemodynamics, postoperative sore throat (POST), nausea, vomiting, dizziness, and agitation.

**Results:**

There was no difference in the baseline data (p > 0.05). There was no significant difference in mouth opening; however, the incidence of swallowing, coughing, body movement, and pharyngeal spasm in group M was lower (p < 0.001), and the proportion of no resistance during LMA insertion was higher (80.5% vs. 21.2%, p < 0.001). The success rate of first-attempt LMA insertion in group M was higher (98.8% vs. 48.2%, p < 0.001), the elapsed time was shorter (16.9 (9.0) vs. 73.0 (91.5) s, p < 0.001), and fewer patients needed additional propofol (1.2% vs. 54.1%, p < 0.001). Blood staining on the LMA surface showed no significant difference, but the postoperative pharyngeal pain score in group M was lower (1.0 (1.0) vs. 2.0 (1.0), p < 0.001). Intraoperative propofol and remifentanil consumption, postoperative dizziness and nausea were lower in group M.

**Conclusion:**

Mivacurium facilitates LMA insertion and reduce intraoperative anesthetic consumption and adverse reactions, such as POST, nausea, and dizziness,so as to benefit the patient.

**Clinical Trial Registration:**

clinicaltrials.gov, identifier ChiCTR2500101122.

## Introduction

The laryngeal mask airway (LMA) is a supraglottic ventilation system. It was invented by Dr. Archie brain in 1983, mainly used for general anesthesia, emergency resuscitation and difficult airway management, with the advantages of simple operation, small trauma, less complications and rapid recovery (Simon and Torp, 2023). However, LMA related complications still deserve clinicians’ attention, such as hypoxia, laryngospasm or bronchospasm, dysphagia or hoarseness (Wong et al., 2012). Among them, postoperative sore throat (POST) is a common complication with incidence of over 20%, which affects the perioperative experience and rehabilitation quality of patients (Jaensson et al., 2014). At present, the main measures to prevent POST after LMA insertion include intravenous analgesics, reducing the pressure of LMA, local anesthetics, etc. (Kim et al., 2021; Altinsoy et al., 2020; Uztüre et al., 2014; Peng et al., 2024) However, it is still necessary to find out more effective and simple measures to reduce POST.

Previous research showed that blood-stained LMA surface was related to POST (Teshome et al., 2024), suggesting that reducing tissue injuries caused by LMA insertion might be a feasible method to reduce POST. Optimizing the anesthesia induction scheme to improve the conditions of LMA insertion could enhance the success rate of LMA insertion (Goertzen et al., 2024), and might reduce mechanical injury. Our previous research showed that the incidence of LMA insertion with moderate or obvious resistance could reach 40% without neuromuscular blocking drugs (Guan et al., 2024). Nevertheless, neuromuscular blocking drugs have residual effects, with risks of respiratory depression, hypopnea, hypoxemia, and retention of carbon dioxide after LMA removal. In particular, LMA is generally performed in minor or day-care surgeries, which may have serious adverse consequences for patients. As a short-acting non-depolarizing neuromuscular blocking drug, mivacurium has the advantages of short duration, rapid recovery, minimal drug accumulation, and fewer neurocardiovascular side effects (Zeng et al., 2017; Wang et al., 2022). It has been proven that mivacurium could facilitate LMA insertion in pediatric day-care surgeries (Ye et al., 2024), however, whether it could show similar clinical application value and improve the short-term prognosis in adults has not been fully explored.

Therefore, we carried out a prospective randomized controlled study to explore whether using mivacurium to general anesthesia induction scheme could facilitate LMA insertion, and reduce the incidence of POST, postoperative nausea and vomiting (PONV), dizziness and other adverse reactions.

## Methods

This single-center, prospective, double-blind, randomized clinical trial was conducted at Weihai Municipal Hospital following the guidelines for the applicable Consolidated Standards of Reporting Trials (CONSORT) (Schulz et al., 2010) for conducting and reporting clinical trials and the Declaration of Helsinki (World Medic al Association, 2013), and was approved by the Ethics Committee of Weihai Municipal Hospital (approval no.2025021). The trial was registered before patient enrollment in the Chinese Clinical Trial Registry (registry no. ChiCTR2500101122, last updated on 21 April 2025). Data from patients undergoing hysteroscopic surgery between April 25 and 31 August 2025, were prospectively collected.

### Patients enrollment and visits

One day before surgery, the researchers preliminarily screened patients who met the research protocol based on the Hospital Information System (HIS) and completed preoperative visits. Patients and their relatives will be fully aware of the potential benefits and risks of participating in the study, and will be informed that they can request withdrawal from the study at any stage without affecting their clinical treatment. Written consent was obtained in a private setting according to institutional guidelines.

The inclusion criteria were as follows: 18–65 years old, hysteroscopic surgery under general anesthesia with LMA, ability to cooperate with communication and complete follow-up indicators, and ability to sign the informed consent form. Exclusion criteria were previous major surgery history, such as cardiac surgery, craniotomy, thoracotomy, etc; emergency surgery; allergy to research medication; patients with mental illness who could not cooperate with follow-up; contraindications for LMA insertion, or predictable difficult airway. Patient participation will be suspended if they withdraw their informed consent form, major perioperative adverse events occur, do not follow the established anesthesia plan, change the surgical method, or perform other surgeries simultaneously.

### Sample size estimation and random grouping

According to our previous study, without the use of neuromuscular blockers, the incidence of mild and obvious resistance in LMA insertion was more than 40% (Guan et al., 2024). It was expected that the proportion could be reduced to less than 20% after the application of mivacurium. The sample size was calculated using PASS (version 21.0.3, NCSS Corporation, USA). With an alpha level of 0.05 and a statistical power level of 0.8, using two-sided tests, the sample size was calculated for 79 patients in each group. Considering a loss to follow-up rate of 10%, 87 patients were required for each group.

Patients were randomized into mivacurium group (group M) or control group (Group C) using the block randomization method, and the interval length of each block was 4. The patients were sorted according to their enrollment time, with a number of 1-4 in each interval. Four numbers corresponding to the patients were extracted from the random number table. Two patients with smaller random numbers were divided into group M, and two patients with larger random numbers were divided into group C. Randomization was performed by an independent researcher. The results were sealed in an opaque envelope until the patient entered the operating room.

The study was double blinded. Neither the researcher nor the patient was aware of the grouping. To ensure the implementation of the blinding method, we set up a dedicated dispensing researcher. After the patient entered the operating room, this researcher opened the grouping envelope, calculated the patient’s mivacurium requirement at a dose of 0.1 mg/kg, and diluted it to 10 mL, or directly extracted 10 mL of saline, and labeled it “research medication”. During anesthesia induction, the drug was injected by an anesthetist at one time for more than 30s.

### Perioperative management

The patients fasted for 8 h with food and 2 h with water, and no preoperative medication was administered before anesthesia. All patients underwent cervical laminaria pretreatment (KL-30, Ken Medical Co., Ltd., Hyogo, Japan) at least 1 h before surgery. Successful cervical pretreatment meant that the laminaria expanded sufficiently, with a diameter of over 7 mm, and the 6 mm diameter cervical dilation rod could smoothly pass through the cervix. After entering the operating room, non-invasive blood pressure, heart rate (HR), electrocardiogram, blood oxygen saturation, end-tidal CO_2_ partial pressure (PetCO_2_), and bispectral index (BIS) were routinely monitored.

Anesthesia induction scheme: Patients in both groups received propofol 2.5 mg/kg + sufentanil 0.25 ug/kg slowly. After consciousness disappeared, the “research medication” was slowly injected at one time. After the eyelash reflex disappeared, a classic laryngeal mask airway (LMA Classic) was inserted for mechanical ventilation. The tidal volume was set at 6–8 mL/kg, the ventilation frequency was 12–18 times/min, positive end-expiratory pressure (PEEP) was not set, and the oxygen concentration was 50%–70%. Patients were treated with the same anesthesia maintenance scheme of propofol 4–12 mg/kg/h + remifentanil 0.05–0.5ug/kg/min, maintaining the BIS between 40–60. Postoperatively, anesthesia recovery was performed in the operating room, and the LMA was removed when the patients could open their eyes, follow the instructions, recover their spontaneous breathing and muscle strength, and their oxygen saturation did not decrease when inhaled. No conventional analgesics or antiemetics were administered postoperatively. According to the patient’s pain, nausea, and vomiting, the anesthesiologist could determine whether to administer the relevant drugs. The patient was transferred to the post-anesthesia care unit (PACU) for continuous monitoring. After the vital signs were stable and the patient had no severe adverse reactions, the was transferred back to the ward. Anesthesia was performed by an anesthesiologist designated by the research team, who had more than 5 years of working experience. All surgeries were performed by the same team.

If the first attempt to insert LMA failed, or the anesthesiologist assessed it was difficult to insert LMA, the anesthesiologist could determine whether additional 30–50 mg of propofol was needed; if LMA insertion failed, tracheal intubation was performed, and the patient was excluded from follow-up. When the hemodynamics fluctuated, the anesthesiologist was responsible for determining whether rescue medication was needed, and the type, dose, and use time of medication were recorded. If LMA displacement occurs during surgery, the anesthesiologist will try to adjust the position of the LMA or deepen the anesthesia. If the position of the LMA could not be adjusted to meet the ventilation demand, tracheal intubation was performed, and the patient was excluded from follow-up. When the patient showed intraoperative body movement, 30–50 mg of propofol was added immediately, and the anesthesiologist determined whether to deepen anesthesia. When other serious adverse events occurred, if they were considered to be related to the study, emergency unblinding was required, and adverse events were recorded and reported according to the procedure.

### Outcomes measurement

All data were collected by the designated researcher of the research group, who would not participate in randomization, blinding, study drug configuration, or data statistical analysis. The primary outcome measure was LMA insertion condition. Referring to Tang et al.‘s research method (Tang et al., 2023), we evaluated the LMA insertion process from different dimensions, including mouth opening, cough, swallowing, head or body motion, laryngospasm, and the overall evaluation. Each index was divided into three levels: level 1 (ideal mouth opening, no cough, swallowing, body movement, laryngospasm, and LMA insertion with no resistance), level 2 (poor mouth opening, mild cough, swallowing, body movement, laryngospasm, and LMA insertion with mild resistance), and level 3 (inability to open the mouth, obvious cough, swallowing, body movement, laryngospasm, and LMA insertion with obvious resistance).

Secondary outcome measures: LMA insertion process: including whether the first attempt and final insertion was successful, the time of attempts, whether additional propofol was required during LMA insertion, the time required for LMA insertion, and parameters of respirator after LMA insertion (tidal volume, peak airway pressure, and plateau pressure); systolic blood pressure (SBP), diastolic blood pressure (DBP), mean blood pressure (MBP) and HR when entering the operating room (T1), after anesthesia induction (T2), after LMA insertion (T3), 5 min after LMA insertion (T4) and 10 min after LMA insertion (T5); whether the LMA was displaced and whether the patient had body movement during the operation; patient recovery evaluation: including anesthesia recovery time, whether there was blood stain on the surface of LMA, POST pain score of throat after LMA removal (using numerical rating scale (NRS), 0–10), Richmond agitation and sedation scale (RASS) (Sessler et al., 2002) after LMA removal, and dizziness, postoperative nausea and vomiting (PONV) (Gan et al., 2020), agitation in PACU.

### Statistical analysis

Statistical analyses were conducted by a designated researcher who was not involved in any part of the study. Missing data were handled using pairwise deletion and data analysis followed the Per-Protocol Set. Continuous data are presented as means and standard deviations, and were analyzed using a t-test for normally distributed variables or the Mann-Whitney U test for nonparametric variables. Categorical data were presented as numbers and analyzed using the χ2 or Fisher’s exact test, as appropriate. Repeated measurement data such as blood pressure and HR were analyzed using repeated-measures analysis of variance (ANOVA), and comparisons of each measurement time were performed using multivariate analysis of variance. Statistical significance was set at p < 0.05. The results were analyzed using IBM SPSS Statistics for Windows (version 27; IBM Corporation, USA).

## Results

**FIGURE 1:**
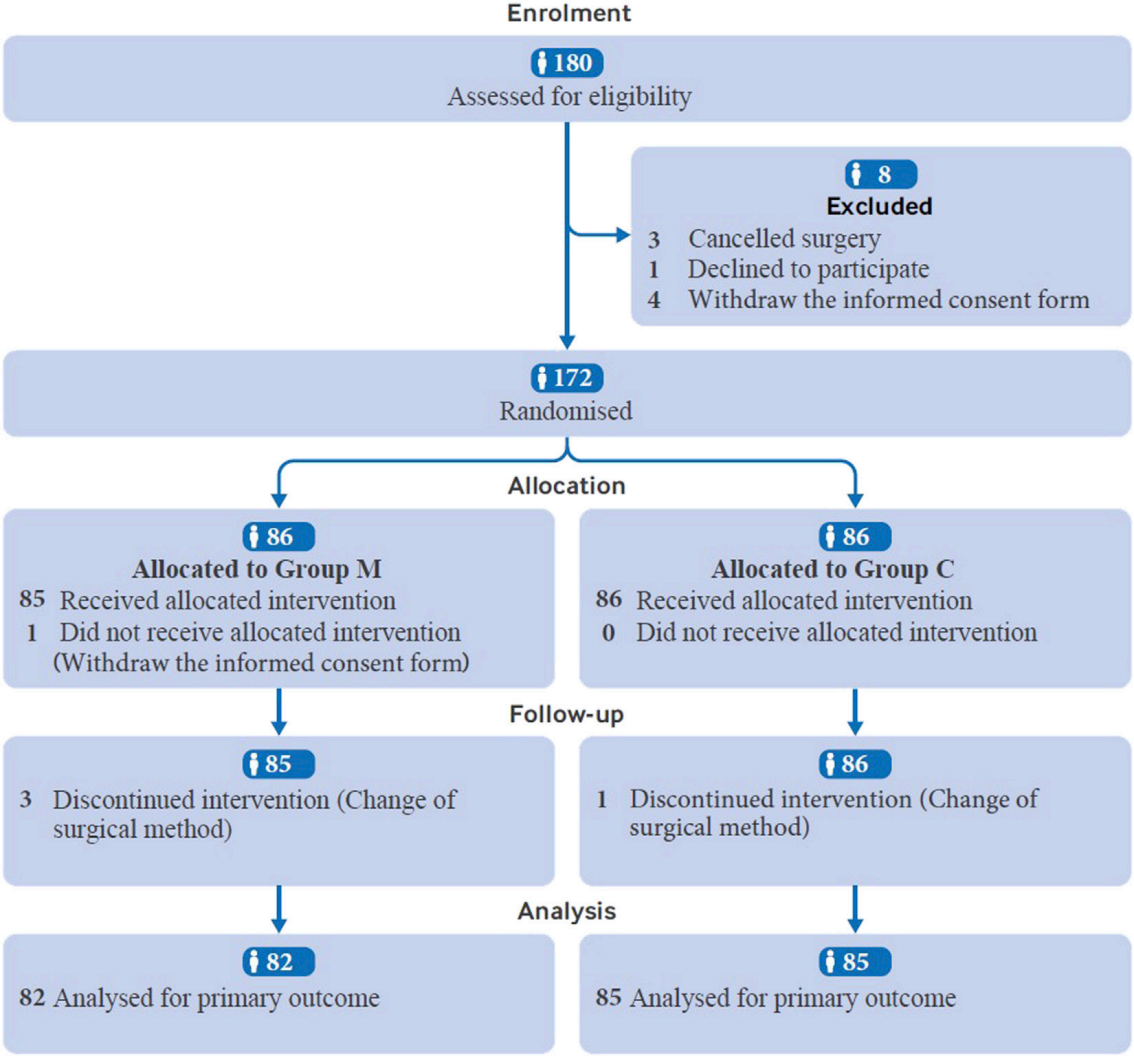
Patient screening, enrollment and randomization.

In total, 180 patients were assessed for eligibility. One patient declined to participate, four withdrew the informed consent form, and three were excluded for surgery cancellation. The remaining 172 patients were randomly assigned to different treatment groups. Four patients in group M were excluded from further analysis because of surgical method change (n = 3) and informed consent form withdrawal (n = 1), while one patient was excluded from group C because of surgical method change. Finally, 167 patients were analyzed, with 82 in Group M and 85 in Group C (Figure 1). The demographic characteristics of the patients, including age, body mass index (BMI), American Society of Anesthesiologists (ASA) physical status classification, Mallampati score, and preoperative examination findings were not significantly different between the two groups (Table 1).

**TABLE 1:** Characteristics of participants before surgery.

Variables	Group M (n = 82)	Group C (n = 85)	p
Age (years)	45.11 ± 8.86	44.15 ± 9.27	0.497
BMI(kg/cm^2^)	24.88 ± 3.93	25.57 ± 4.33	0.286
ASA (n (%))			0.837
I	3 (3.7%)	2 (2.4%)	
II	79 (96.3%)	82 (96.5%)	
III	0 (0.0%)	1 (1.2%)	
Mallampati score (n (%))			0.486
I	48 (58.5%)	51 (60.0%)	
II	34 (41.5%)	34 (40.0%)	
Comorbidities (n (%))			
Hypertension	9 (11.0%)	6 (7.1%)	0.270
Diabetes	4 (4.9%)	4 (4.7%)	0.620
Coronary heart disease	0 (0.0%)	2 (2.4%)	0.258
Duration of Anesthesia (min)	31.50 (12.50)	30.00 (12.00)	0.617
Duration of surgery (min)	20.00 (13.00)	19.00 (10.00)	0.532
Surgical site (n (%))			0.152
Endometrium	49 (59.8%)	47 (55.3%)	
Both endometrium and cervical canal	27 (32.9%)	22 (25.9%)	
Cervical canal	3 (3.7%)	10 (11.8%)	
Others	3 (3.7%)	6 (7.1%)	
Laboratory examination			
RBC (10^9^/L)	4.35 ± 0.43	4.37 ± 0.41	0.715
WBC (10^9^/L)	5.98 ± 1.70	5.85 ± 1.39	0.567
HGB (g/L)	124.50 (27.50)	128.50 (28.75)	0.318
PLT (10^9^/L)	272.17 ± 74.53	257.04 ± 67.58	0.172
PT (s)	11.47 ± 0.67	11.40 ± 0.68	0.462
APTT (s)	27.95 ± 2.79	27.85 ± 2.99	0.832
ALT (U/L)	13.80 (7.70)	14.30 (12.45)	0.855
AST (U/L)	17.25 (5.78)	16.10 (6.83)	0.053
BUN (mmol/L)	4.20 (1.70)	4.20 (1.35)	0.985
Scr (μmol/L)	52.00 (10.00)	52.50 (13.50)	1.000
ECG examination (n (%))			0.317
Normal	55 (67.1%)	53 (62.4%)	
Abnormal (Simon and Torp, 2023)	27 (32.9%)	32 (37.6%)	
Chest radiograph (n (%))			0.520
Normal	62 (75.6%)	65 (76.5%)	
Abnormal (Wong et al., 2012)	20 (24.4%)	20 (23.5%)	

Abnormal electrocardiogram includes sinus tachycardia, sinus bradycardia, sinus arrhythmia, atrial premature beats, ventricular premature beats, etc.; 2: Abnormal chest radiography includes increased lung markings and pulmonary nodules.

Abbreviations: RBC: red blood cell count; WBC: white blood cell count; HGB: hemoglobin; PLT: platelet; PT: prothrombin time; APTT: activated partial thromboplastin time; ALT: alanine aminotransferase; AST: aspartate aminotransferase; ALB: albumin; TB: total bilirubin; DB: direct bilirubin; BUN: blood urea nitrogen; Scr: Serum Creatinine.

There was no significant difference in mouth opening between the two groups. But for patients in group M, the proportion without swallowing action was higher (97.6% vs. 35.3%, p < 0.001), the proportion without cough reaction was higher (97.6% vs. 56.8%, p < 0.001), the proportion without head or body motion was higher (98.8% vs. 74.1%, p < 0.001), the incidence of laryngospasm reaction was lower (3.7% vs. 67.1%, p < 0.001), and the proportion of LMA insertion evaluated as “no resistance” was higher (80.5% vs. 21.2%, p < 0.001). At the same time, the success rate of first-attempt LMA insertion in group M was higher (98.8% vs. 48.2%, p < 0.001), the LMA insertion time was shorter (16.9 (9.0) vs. 79.0 (91.5) s, p < 0.001), and fewer patients required additional propofol during LMA insertion (1.2% vs. 54.1%, p < 0.001) (Table 2).

**TABLE 2:** Analysis of LMA insertion.

Variables	Group M (n = 82)	Group C (n = 85)	p
Grading conditions for LMA Insertion (n (%))			
Mouth opening			0.141
Full	68 (82.9%)	60 (70.6%)	
Partial	13 (15.9%)	23 (27.1%)	
None	1 (1.2%)	2 (2.4%)	
Swallowing			<0.001*
Nil	80 (97.6%)	30 (35.3%)	
Mild	2 (2.4%)	53 (62.4%)	
Severe	0 (0%)	2 (2.4%)	
Coughing			<0.001*
Nil	80 (97.6%)	48 (56.8%)	
Mild	1 (1.2%)	34 (40.0%)	
Severe	1 (1.2%)	3 (3.5%)	
Head or body motion			<0.001*
Nil	81 (98.8%)	63 (74.1%)	
Mild	1 (1.2%)	19 (22.4%)	
Severe	0 (0%)	3 (3.5%)	
Laryngospasm			<0.001*
Nil	79 (96.3%)	28 (32.9%)	
Mild	3 (3.7%)	52 (61.2%)	
Severe	0 (0%)	5 (5.9%)	
Ease of LMA insertion			<0.001*
No resistance	66 (80.5%)	18 (21.2%)	
Mild resistance	16 (19.5%)	59 (69.4%)	
Obvious resistance	0 (0%)	8 (9.4%)	

Abbreviations: LMA, laryngeal mask airway.

**FIGURE 2:**
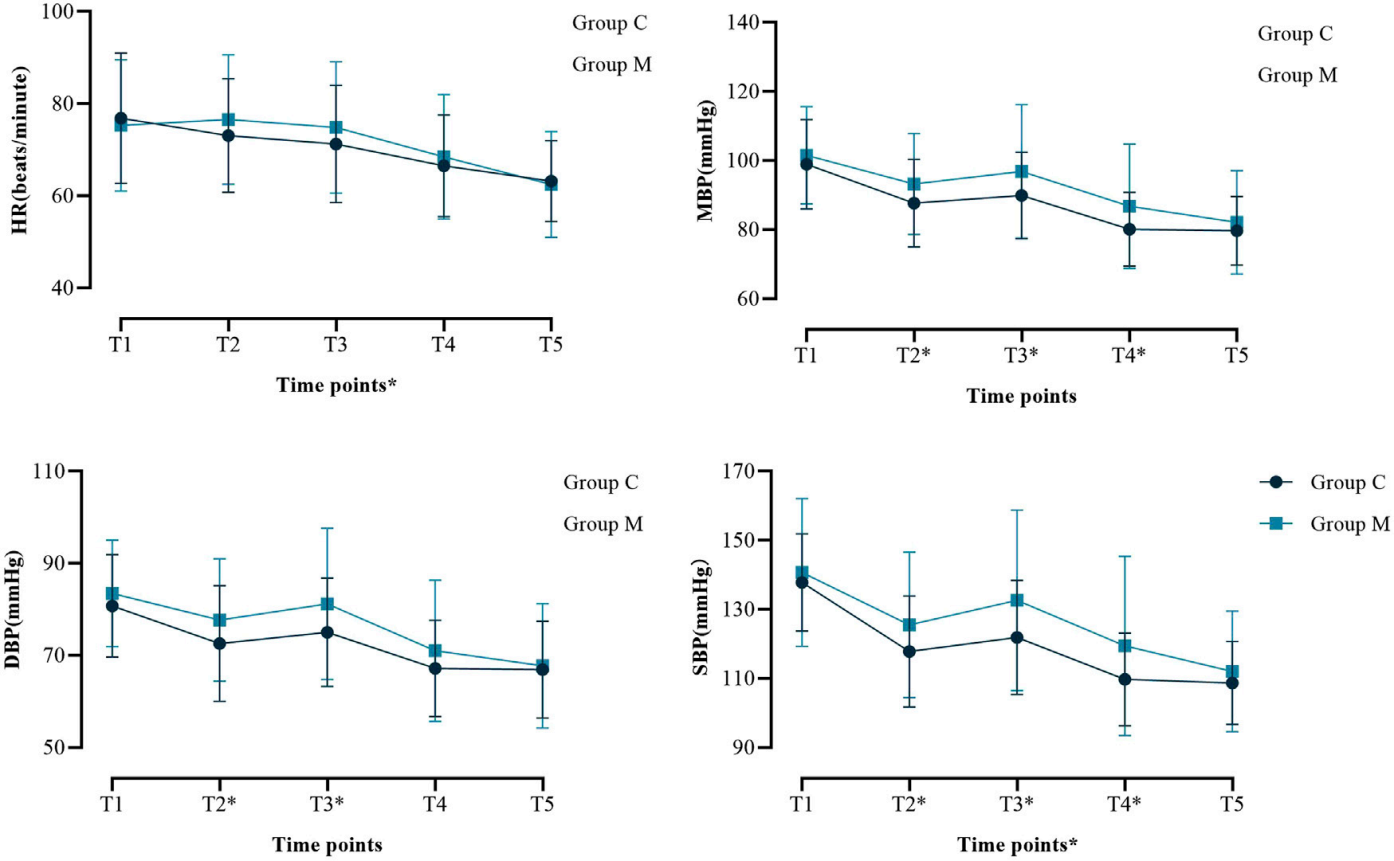
Perioperative hemodynamic outcome indicators. Systolic blood pressure (SBP); Diastolic blood pressure (DBP); Mean Blood Pressure (MBP); Heart rate (HR). *P < 0.05 at this time point. Abbreviations: SBP, systolic blood pressure; DBP, diastolic blood pressure; MBP, Mean Blood Pressure; HR, heart rate; T1, after the patient entered the operating room; T2, after induction of anesthesia; T3, immediately after LMA insertion; T4, 5 minutes after LMA insertion; T5, 10 minutes after LMA insertion.

There were no significant differences in peak airway pressure or tidal volume between the two groups (Supplementary Table S1). There was no significant difference in the incidence of intraoperative body movement reaction and LMA displacement (Supplementary Table S1), but the intraoperative consumption of propofol (0.082 (0.04) vs. 0.090 (0.04) mg/kg/min, p = 0.045) and remifentanil (0.057 ± 0.015 vs. 0.069 ± 0.021 μg/kg/min, p < 0.001) in group M was lower than that in the control group (Table 3). After LMA removal, there was no significant difference in the incidence of blood staining on the LMA surface; however, the NRS score of POST in group M was lower (1.00 (1.00) vs. 2.00 (1.00), p < 0.001) (Table 3). Intraoperative blood pressure and HR were generally within the normal range, although there were statistical differences at several time points (Figure 2; Supplementary Table S2). The recovery time of group M was slightly longer than control group (6.00 (2.00) vs. 5.00 (3.00) min, p = 0.048), and the proportion of patients with RASS score of −1 or below after recovery was higher (43.9% vs. 21.2%, p = 0.002) (Table 3). There was no significant difference in the incidence of agitation and vomiting after recovery; however, the incidence of postoperative nausea (4.9% vs. 15.3%, p = 0.039) and dizziness (35.4% vs. 57.6%, p = 0.005) was lower in group M than in the control group (Table 3). No postoperative respiratory depression occurred in any patient (data not shown).

**TABLE 3:** Analysis of secondary outcome indicators.

Variables	Group M (n = 82)	Group C (n = 85)	p
First-time insertion success (n (%))	81 (98.8%)	41 (48.2%)	<0.001*
Insertion attempts (n (%))			<0.001*
1	81 (98.8%)	41 (48.2%)	
2	1 (1.2%)	41 (48.2%)	
3	0 (%)	3 (3.5%)	
Propofol supplementation (n (%))	1 (1.2%)	46 (54.1%)	<0.001*
LMA insertion time(s)	16.90 (9.00)	73.00 (91.50)	<0.001*
Intraoperative anesthesia consumption			
Propofol (mg/kg/min)	0.082 (0.04)	0.090 (0.04)	0.045*
Remifentanil (ug/kg/min)	0.057 ± 0.015	0.069 ± 0.021	<0.001*
Evaluation of anesthesia recovery			
Anesthesia recovery time (min)	6.00 (2.00)	5.00 (3.00)	0.048*
NRS of POST	1.00 (1.00)	2.00 (1.00)	<0.001*
Blood stain on LMA surface (n (%))	7 (8.5%)	16 (18.8%)	0.072
RASS Score			0.002*
−2	6 (7.3%)	0 (0.0%)	
−1	30 (36.6%)	18 (21.2%)	
0	44 (53.7%)	62 (72.9%)	
1	2 (2.4%)	5 (5.9%)	
Agitation (n (%))	0 (0%)	5 (5.9%)	0.059
Nausea (n (%))	4 (4.9%)	13 (15.3%)	0.039
Vomiting (n (%))	2 (2.4%)	0 (0.0%)	0.240
Dizziness (n (%))	29 (35.4%)	49 (57.6%)	0.005*

Abbreviations: LMA, laryngeal mask airway; NRS, numerical rating scale; POST, postoperative sour throat; RASS, richmond agitation and sedation scale.

## Discussion

Hysteroscopy is a minor surgery that can be performed in an outpatient surgery model or patients can be discharged within a short time after surgery. Therefore, when formulating an anesthesia scheme, clinicians should fully consider adverse reactions and residual effects to avoid delayed discharge or respiratory depression after leaving the hospital, which might threaten patient safety. With this in mind, some anesthesiologists should be very careful when using neuromuscular blocking drugs. However, non-use of neuromuscular blocking drugs may lead to resistance during LMA insertion and increase the risk of throat injury. Therefore, we chose mivacurium, a short-acting neuromuscular blocking drug, and more conservatively, half of the conventional induction dose in this study. Our results showed that mivacurium could still improve the conditions of LMA insertion, even with a relatively conservative dose. However, two problems remain to be addressed. First, mivacurium did not improve mouth opening. This may be because the degree of mouth opening is not completely related to muscle relaxation. Studies have shown that age, sex, BMI, and other factors could also affect the mouth opening of patients (Apfelbaum et al., 2022; Gallagher et al., 2004; Aliya et al., 2021). This might also be the reason why 19.5% of the patients in group M still had mild resistance to LMA insertion. Second, a few patients in group M still had varying degrees of body movement and laryngospasm, although the proportion was very low. This showed that the mivacurium dose used in the study could not achieve satisfactory muscle relaxation in all patients. However, it also showed that the dose we selected could meet the clinical needs of rapid metabolism.

Meanwhile, our research indicates that patients in group M had a higher success rate in the first attempt of LMA insertion, required less additional propofol, and consumed less total time for insertion process. We have reason to believe that this is due to the improvement of the conditions for LMA insertion, where the patient’s pharyngeal muscles have better relaxation and less resistance. Other studies have also made similar findings. A study conducted in Turkey showed that improving the placement conditions of LMA can accelerate the speed of LMA placement and reduce the hemodynamic fluctuation (Çakır et al., 2025). Zheng et al.'s (Zheng et al., 2025) meta-analysis found that insertion of LMA using a laryngoscope-guided technique can improve fibrotic staging, oxidative lean pressure, and success rate for the first attempt of LMA insertion. Therefore, improving the implantation conditions during LMA implantation still has positive clinical significance. At present, although there are studies exploring the clinical effects of a single intervention, there is no standard operating procedure for improving LMA implantation conditions, or exploring whether combining multiple intervention methods can have a better effect on improving LMA implantation conditions. Therefore, further research is still necessary.

We found that patients in group M had a lower intraoperative demand for propofol and remifentanil. We could not directly analyze the reasons based on our existing research results. We suspected that after the application of mivacurium, the LMA insertion was smoother, irritation to the throat was decreased, and tolerance to mechanical ventilation was improved; thus, the demand for anesthetic drugs was reduced. A randomized controlled study in patients undergoing colorectal laparoscopic surgery showed that compared with moderate muscle relaxant, deep muscle relaxant could reduce the consumption of remifentanil from 494 (392–618) ug/h to 348 (228–472)ug/h, indicating that the degree of muscle relaxant might affect the consumption of sedatives and analgesics during anesthesia (Morisson et al., 2024). The research of George et al. showed similar results with ours (George et al., 2017). They used 2 mg/kg propofol+2ug/kg fentanyl + placebo, 0.1 mg/kg or 0.25 mg/kg of succinylcholine for anesthesia induction before LMA insertion. They also found that the application of succinylcholine could reduce the total consumption of propofol during anesthesia, although the authors did not point out the possible reasons for this phenomenon.

In terms of anesthesia recovery, we found that there was no significant difference in blood staining on the surface after LMA removal, although the incidence in the control group was higher. This suggests that neuromuscular blockers might not be the only factor to reduce LMA-related pharyngeal injury, and the proficiency of physicians, LMA structure, and cuff pressure are all related to LMA pharyngeal injury (Li et al., 2021). But the VAS score of POST in group M was lower. This shows that the application of mivacurium to improve the conditions of LMA insertion was still an effective method for reducing POST. Research have found that the incidence of POST after LMA insertion is around 20%, and is not related to demographic variables (such as age, sex, and BMI) (Farazmehr et al., 2021). Therefore, this might be related to the alleviation of mucosal and tissue contusions caused by the reduction of resistance during LMA insertion, relaxation of pharyngeal muscles to reduce LMA compression, and fewer times of LMA insertion attempts to reduce mechanical damage to the throat. The anesthesia recovery time in group M was slightly longer than that in the control group. Although the difference was statistically significant, considering that the recovery time of the two groups was within 10 min, this extension of the recovery time did not increase the clinical burden. However, we noticed that the proportion of patients with RASS score of −1 and below in group M was higher than that in the control group. Interestingly, these two differences occurred on the premise that the consumption of anesthetic and analgesic drugs in group M was lower. We believe that this might be because the degree of POST in group M was lower and the tolerance to LMA was better; therefore, the recovery time of patients was slightly prolonged, and the degree of postoperative sedation was slightly deeper in group M. In terms of short-term postoperative adverse reactions, dizziness was still a high-incidence complication, but the incidence in group M was lower, and so was postoperative nausea. Jiang et al. (Jiang et al., 2025) found that vitamin C or dexamethasone can reduce postoperative inflammatory factors levels and decrease the incidence of PONV. Therefore, we speculated that the decrease in postoperative nausea in group M mighty be related to increased tolerance to LMA, reduced pharyngeal damage, and decreased inflammation and stress levels, but further research is needed to confirm this. The mechanism of postoperative dizziness is complex, and multiple factors could be related to its occurrence such as gender, age, perioperative anesthesia, and analgesic drugs (Zhou et al., 2025). Indeed, further research is needed to explore its mechanisms and effective prevention and control strategies. Although there was no difference in postoperative agitation, we found that all patients diagnosed with postoperative agitation were in the control group. Many studies have shown that inflammatory factors and pain are related to postoperative agitation (Imai et al., 2023; Wei et al., 2021; Wang et al., 2023), thus we speculated that this might be related to the emphasis on POST in the control group. Although there were differences in anesthesia recovery time, we believe that these differences do not have significant clinical significance. The anesthesia recovery time of both groups is within the clinically acceptable range and would not increase any additional medical burden.

Our study has some limitations. First, it was a single-center study, the sample size was small, and all patients we included were women; therefore, whether our research conclusion is universal still needs more research and discussion. It is not yet possible to infer whether the same clinical effects will be observed in populations such as males, the elderly, and children. Second, we set a smaller dose of mivacurium, which is half of the anesthesia induction dose, and we did not conduct train of four (TOF) monitoring. Therefore, we lacked quantitative data to judge the degree of muscle relaxation, and we could not compare whether this dose is more conducive to improving the LMA insertion conditions; third, we did not measure the inflammatory index, oxidative stress index, and other laboratory indicators, so our study is still unable to explore the mechanism of mivacurium in reducing POST and other adverse reactions in patients with LMA; fourth, we did not conduct long-term follow-up of patients, so whether our anesthesia scheme can improve the long-term prognosis still needs further observation; fifth, limited by the study sample, we did not conduct subgroup analysis on patients (stratified by age), so we could not find the most suitable population for this anesthesia scheme. These limitations will be explored in further research to investigate the clinical application and mechanism of the benefit of mivacurium in LMA.

## Conclusion

The application of low-dose mivacurium in the anesthesia induction scheme of hysteroscopic surgery under LMA anesthesia can improve the LMA insertion conditions, increase the first attempt success rate, reduce the time required of LMA insertion, reduce the intraoperative consumption of anesthetic drugs, and reduce postoperative adverse reactions such as POST, dizziness, and nausea, which could benefit patients in short surgeries under LMA anesthesia.

## Data Availability

The raw data supporting the conclusions of this article will be made available by the authors, without undue reservation.
