# Supplementary material for: Low-dose mivacurium facilitates laryngeal mask airway insertion in patients undergoing hysteroscopic surgery: a prospective, single-center, double-blind randomized controlled trial
Source: Front Pharmacol. 2025 Oct 17;16:1700175. doi: 10.3389/fphar.2025.1700175 (PMC12575122; doi:10.3389/fphar.2025.1700175)
Supplement: Supplementary file 1 [file Table1.docx]

**Table S1 Intraoperative ventilator parameters and adverse reactions**

|  | **Group M**  **(n=82)** | **Group C**  **(n=85)** | **p** |
| --- | --- | --- | --- |
| **Intraoperative ventilator parameters** |  |  |  |
| Peak airway pressure(mmHg) | 13.00(3.00) | 14.00(2.50) | 0.076 |
| Plateau pressure(mmHg) | 13.00(2.00) | 14.00(3.00) | 0.031* |
| Tidal volume(ml） | 473.00(74.25) | 482.00(88.50) | 0.877 |
| Intraoperative body movement(n(%)) | 5(6.1%) | 2(2.4%) | 0.271 |
| Intraoperative LMA displacement(n(%)) | 5(6.4%) | 8(9.4%) | 0.566 |
