# Supplementary material for: Low-dose mivacurium facilitates laryngeal mask airway insertion in patients undergoing hysteroscopic surgery: a prospective, single-center, double-blind randomized controlled trial
Source: Front Pharmacol. 2025 Oct 17;16:1700175. doi: 10.3389/fphar.2025.1700175 (PMC12575122; doi:10.3389/fphar.2025.1700175)
Supplement: Supplementary file 2 [file Table2.docx]

**Table S2 Perioperative hemodynamic outcome indicators**

|  | **Group M**  **(n=82)** | **Group C**  **(n=85)** | **p** |
| --- | --- | --- | --- |
| SBP（mmHg） |  |  | 0.013*^1^ |
| T1 | 140.73±21.46 | 137.84±14.13 | 0.30 |
| T2 | 125.51±21.02 | 117.85±16.12 | 0.009* |
| T3 | 132.61±26.17 | 121.87±16.50 | 0.002* |
| T4 | 119.41±26.00 | 109.75±13.44 | 0.003* |
| T5 | 112.04±17.50 | 108.72±11.99 | 0.153 |
| DBP（mmHg） |  |  | 0.079^1^ |
| T1 | 83.46±11.57 | 80.75±11.11 | 0.124 |
| T2 | 77.69±13.29 | 72.59±12.55 | 0.012* |
| T3 | 81.22±16.46 | 75.04±11.78 | 0.006* |
| T4 | 71.04±15.32 | 67.21±10.48 | 0.061 |
| T5 | 67.77±13.51 | 66.94±10.51 | 0.659 |
| MBP（mmHg） |  |  | 0.093^1^ |
| T1 | 101.49±14.12 | 98.89±13.03 | 0.219 |
| T2 | 93.24±14.62 | 87.66±12.66 | 0.009* |
| T3 | 96.84±19.33 | 89.91±12.54 | 0.006* |
| T4 | 86.80±18.06 | 80.12±10.68 | 0.004* |
| T5 | 82.13±15.02 | 79.64±9.95 | 0.207 |
| HR（beats per minute） |  |  | 0.002^1^* |
| T1 | 75.30±14.30 | 76.84±14.17 | 0.485 |
| T2 | 76.61±14.09 | 73.06±12.37 | 0.085 |
| T3 | 74.85±14.30 | 71.27±12.75 | 0.089 |
| T4 | 68.48±13.53 | 66.53±11.05 | 0.309 |
| T5 | 62.35±11.51 | 63.21±8.80 | 0.588 |

Note: 1. The p-value here refers to the p-value of the interaction effect of the intra subject factor * grouping in the intra subject effect test
